# Are Pain-Related Fears Mediators for Reducing Disability and Pain in Patients with Complex Regional Pain Syndrome Type 1? An Explorative Analysis on Pain Exposure Physical Therapy

**DOI:** 10.1371/journal.pone.0123008

**Published:** 2015-04-28

**Authors:** Karlijn J. Barnhoorn, J. Bart Staal, Robert T. M. van Dongen, Jan Paul M. Frölke, Frank P. Klomp, Henk van de Meent, Han Samwel, Maria W. G. Nijhuis-van der Sanden

**Affiliations:** 1 Scientific Institute for Quality of Healthcare, Radboud university medical center, Nijmegen, The Netherlands; 2 Department of Anaesthesiology, Pain and Palliative Care, Radboud university medical center, Nijmegen, The Netherlands; 3 Department of Surgery, Radboud university medical center, Nijmegen, The Netherlands; 4 Department of Orthopaedics, Section Physical Therapy, Radboud university medical center, Nijmegen, The Netherlands; 5 Department of Rehabilitation, Radboud university medical center, Nijmegen, The Netherlands; 6 Department of Medical Psychology, Radboud university medical center, Nijmegen, The Netherlands; The James Cook University Hospital, UNITED KINGDOM

## Abstract

**Objective:**

To investigate whether pain-related fears are mediators for reducing disability and pain in patients with Complex Regional Pain Syndrome type 1 when treating with Pain Exposure Physical Therapy.

**Design:**

An explorative secondary analysis of a randomised controlled trial.

**Participants:**

Fifty-six patients with Complex Regional Pain Syndrome type 1.

**Interventions:**

The experimental group received Pain Exposure Physical Therapy in a maximum of five treatment sessions; the control group received conventional treatment following the Dutch multidisciplinary guideline.

**Outcome measures:**

Levels of disability, pain, and pain-related fears (fear-avoidance beliefs, pain catastrophizing, and kinesiophobia) were measured at baseline and after 3, 6, and 9 months follow-up.

**Results:**

The experimental group had a significantly larger decrease in disability of 7.77 points (95% CI 1.09 to 14.45) and in pain of 1.83 points (95% CI 0.44 to 3.23) over nine months than the control group. The potential mediators pain-related fears decreased significantly in both groups, but there were no significant differences between groups, which indicated that there was no mediation.

**Conclusion:**

The reduction of pain-related fears was comparable in both groups. We found no indication that pain-related fears mediate the larger reduction of disability and pain in patients with Complex Regional Pain Syndrome type 1 treated with Pain Exposure Physical Therapy compared to conventional treatment.

**Trial registration:**

International Clinical Trials Registry NCT00817128

## Introduction

Complex Regional Pain Syndrome type 1 (CRPS-1) is a debilitating condition that develops spontaneously or after physical injury, and is characterized by pain and sensory, autonomic, motor, and/or trophic changes [1]. The exact cause of CRPS-1 is still not fully understood, but various pathophysiologic pathways have been identified that contribute to the heterogeneous clinical presentation of the disorder [2].

Aberrant neurogenic inflammation and vasomotor dysfunction cause increased temperature, skin reddening, protein extravasation, oedema, and augmented nociceptive stimulation, whereas maladaptive cortical reorganization contributes to experienced pain and motor dysfunction [2]. The degree of cortical reorganization is found to be directly related to intensity of pain [3].

The role of psychological factors has also been considered, both in the development of CRPS-1 and in its progression [4]. It has been shown that, in chronic musculoskeletal pain, patients’ beliefs about their pain are a disabling factor, and that pain-related fear and associated avoidance behaviours contribute to the development of chronic pain-related disability [5]. Similar associations are suggested for patients with CRPS-1 [6].

**Fig 1:**
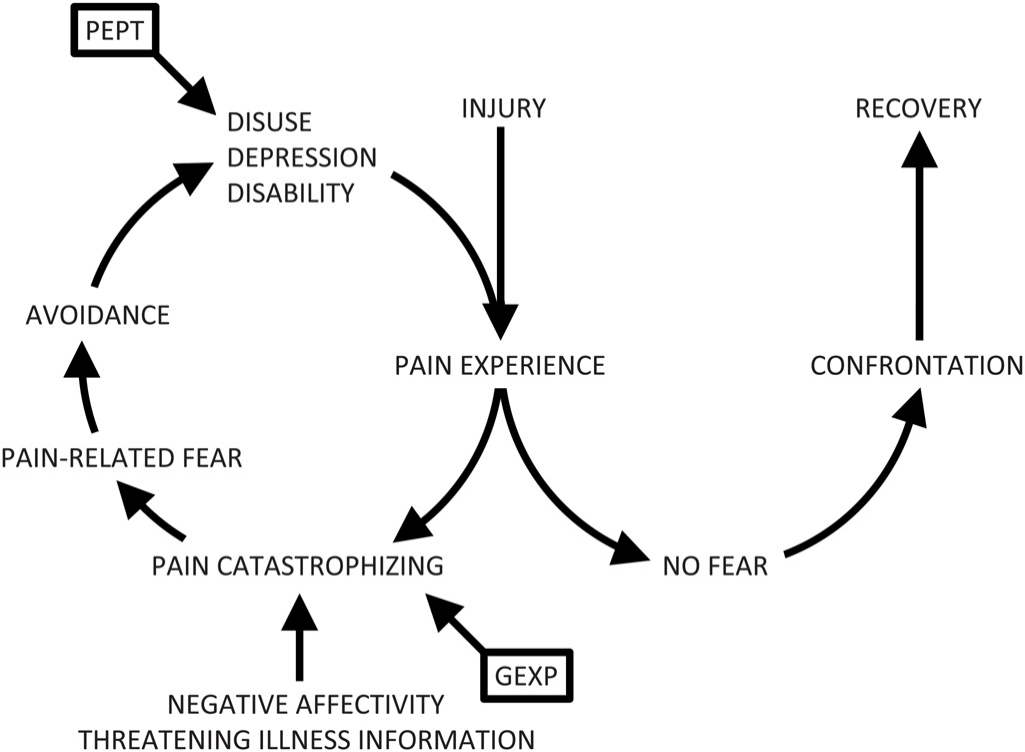
The fear-avoidance model, showing the targets of Pain Exposure Physical Therapy and graded exposure treatment. In this model, pain catastrophizing, pain-related fear and avoidance are thought to be mediators for the treatment of disability and consequently pain. Reproduced from Vlaeyen and Linton [7]. PEPT = Pain Exposure Physical Therapy. GEXP = Graded Exposure treatment.

One of the models that could explain the associations between fear and CRPS-1 is the fear-avoidance model (Fig 1) [7]. According to this model, pain-related fear influences the development and maintenance of pain-related disability [8]. Pain may lead to catastrophic thoughts, which lead to fear and avoidance of movements. In turn, this avoidance leads to disability and increase of pain [9].

In a meta-analysis about pain-related fear and disability in patients with acute and chronic pain, Zale et al. concluded that pain-related fear might be an important target for treatments intended to reduce pain-related disability [10]. Looking at the fear-avoidance model, it could be expected that interfering with the vicious circle by reducing catastrophic thoughts and fear of pain would decrease disability and pain, as is shown with graded exposure therapy (GEXP) [11]. GEXP is based on the fear-avoidance model and, using a graded hierarchy of fear-eliciting situations, it modifies the meaning patients attach to their pain. Reduction of catastrophic thoughts and fear of pain with graded fear exposure is associated with regaining functional abilities in patients with CRPS-1 [8,11,12].

According to the principles of the fear-avoidance model, pain catastrophizing and pain-related fear have to be treated first, in order to restore functional abilities (Fig 1). However, it may be possible that a more functionally directed approach, with the focus on increased motor activity and normal use of the affected limb in daily life, could also lead to improved patient outcomes. Ten years ago, the practices of a Macedonian woman, Ms Shinka, caught the attention of two Dutch physicians. She literally ignored the patients’ expressions of pain and their fear of someone touching their affected limb, and started moving it passively. With a remarkable result: she appeared to be able to considerably decrease pain and improve function in patients with treatment-resistant CRPS-1. [13]. As a derivative of her treatment, Pain Exposure Physical Therapy (PEPT) was developed, in which therapists no longer focus their attention on pain or fear, but rather on physical activities and function [14,15].

PEPT is a patient-centred physical therapy, tailored towards improving activities in daily life, using progressive-loading exercises, desensitization and “self-forced” use, in which patients have to encourage themselves to use their affected extremity, both during treatment exercises and in daily activities, without the use of medication. Whereas graded exposure therapy focuses on fear, PEPT puts disuse at the centre. It takes patients to their physiological boundaries to regain normal functional abilities. Ek et al. studied PEPT in a cohort of 106 adult patients with long-lasting CRPS-1. Function improved in 90% of patients, and full functional recovery was observed in 46% of patients [14]. In a multiple single-case study on patients with CRPS-1, Van de Meent et al. demonstrated the safety and possible effectiveness of PEPT [15].

In Dutch clinical practice, clinicians usually treat patients with CRPS-1 with conventional therapy, following the national guideline, which is based on systematic reviews of the available studies at the time of conception [16]. The guideline includes pharmacological interventions with various drugs to decrease pain, and intensive pain-contingent physical therapy. Conventional treatment is mainly focused at decreasing pain, where pain is regarded as a sign of physical overload. By decreasing pain with (usually) pharmacological interventions, the physical capacity is increased with pain-contingent physical therapy.

Recently, we performed a randomized controlled trial (RCT), comparing the effectiveness of PEPT and conventional treatment in patients with CRPS-1, the PEPTOC trial (Pain Exposure Physical Therapy Or Conventional; paper submitted). The primary analysis showed that only the active range of motion improved significantly more after PEPT than after conventional treatment. Further per protocol analysis, however, showed an additional larger reduction of disability and pain in patients treated with PEPT than patients treated conventionally.

Following the results of the multiple single-case study [15] and the PEPTOC trial, we hypothesized that reducing fear is not a prerequisite for reducing disability and pain in patients with CRPS-1, and that treatment does not primarily need to focus on reduction of fear or pain to be effective. To explore the working mechanism of PEPT in more detail, we investigated whether pain-related fears were mediators of the effect of Pain Exposure Physical Therapy on disability and pain in patients with CRPS-1.

## Methods

### Design

The PEPTOC trial was designed as a randomized controlled trial with blinding of the assessor. We collected data at baseline and after three, six, and nine months follow-up [17]. The primary results of the trial will be published elsewhere. The current study is an explorative mediation analysis of the PEPTOC trial.

### Participants

Inclusion criteria for the PEPTOC trial were 1) positive scoring on the research diagnostic criteria for CRPS as proposed by Harden et al. (shown below) [18], 2) age between 18 and 80 years, and 3) first assessment between 3 and 24 months after the inciting event. Criteria for exclusion were 1) CRPS-1 in more than one extremity, 2) relapse of CRPS-1, 3) pregnancy or lactation, and 4) prior sympathectomy of the affected limb.

Diagnostic CRPS criteria [18]

1. Continuing pain, which is disproportionate to any inciting event;
2. At least one symptom in each of the four following categories;
  Sensory: reports of hyperalgesia and/or allodynia
  Vasomotor: reports of temperature asymmetry, skin color changes and/or skin color asymmetry
  Sudomotor/edema: reports of edema, sweating changes and/or sweating asymmetry
  Motor/trophic: reports of decreased range of motion, motor dysfunction and/or trophic changes
3. At least one sign in two or more of the following categories:
  Sensory: evidence of hyperalgesia and/or allodynia
  Vasomotor: evidence of temperature asymmetry (>1°C), skin color changes and/or skin color asymmetry
  Sudomotor/edema: evidence of edema, sweating changes and/or sweating asymmetry
  Motor/trophic: evidence of decreased range of motion, motor dysfunction and/or trophic changes
4. There is no other diagnosis that better explains the signs and symptoms.

### Interventions

After baseline measurements, patients received either the experimental treatment Pain Exposure Physical Therapy (PEPT) or the conventional control treatment (CONV).

#### Experimental treatment

At the start of treatment with PEPT all medication aimed at CRPS-1, including analgesics, was stopped, as use of medication is not part of the treatment. PEPT was conducted by two physical therapists together. Patients received thorough education about their condition, the working mechanism of pain (acute versus chronic, central pain sensation and memory) and pain-related avoidance behaviour, and the rationale and content of PEPT. Therapists explained that pain is not a sign of injury, but rather a “false warning sign”: pain does hurt, but does not mean harm.

Therapists explained that they acknowledge that the patients might temporarily experience pain, but ignored further expressions of pain and taught the patients and their spouses to do the same. The therapists explained that because of improved function, the pain would eventually diminish [14,15,17].

After thorough instruction about PEPT, the patients, along with their spouses, set their personal, functional treatment goals. The therapists acted mainly as instructors and coaches, and confirmed and rewarded progression with positive feedback.

The therapists confidently touched the affected limb and passively moved it beyond the active range of motion. They then stimulated the patients and their spouses to do the same. They convinced the patients that moving and touching the affected limb is safe.

Further treatment consisted of progressive-loading exercises, focused on specific daily activities and quality of movement, and other regular physical therapy techniques, like muscle strength training and joint mobility exercises. Patients learned how to decrease their skin sensitivity for touch and pressure by performing self-massage and “self-forced” use of the affected extremity during exercises and in daily activities. Patients had to perform the exercises at home and incorporate them in their daily life, whereas their spouses had to ensure that the exercises were performed correctly. Spouses were instructed to ignore the patients’ pain behaviour and change their protective role into a motivating and coaching one.

PEPT consists of a maximum of five treatment sessions and is time-contingent, in contrast to the usual pain-contingent physical therapy. The content and the number of treatment sessions are not influenced by experienced pain. More details on Pain Exposure Physical Therapy have been reported elsewhere [14,15,17].

#### Control treatment

Conventional treatment (CONV) was based on the current Dutch national guideline and included pharmacological interventions combined with physical therapy in a pain-contingent manner [16]. The recommendations in this guideline result from a large multidisciplinary consensus meeting and systematic reviews available at the time of conception in 2006. Conventional physical therapy was focused on controlling pain, using movements within pain limits, mild exposure and increasing the capacity to perform daily activities step by step in a pain-contingent manner. Pharmacological treatment included the use of analgesics in a step-up procedure in accordance with the WHO pain ladder; free radical scavengers including dimethylsulphoxide 50% ointment (DMSO) and N-acetylcysteine; calcium channel blockers; and ketanserine. Patients presenting with allodynia or hyperalgesia were given gabapentin, amitriptyline, or carbamazepine. Dystonia, myoclonia, and muscle spasms were treated with baclofen, diazepam, or clonazepam. In the case of cold skin, the anaesthesiologist would prescribe vasodilating drugs such as verapamil, ketensin, and pentoxiphylline. In case of insufficient clinical effect, sympathetic blockade, transcutaneous electrical nerve stimulation, or spinal cord stimulation was considered [16].

### Outcome measures

The outcomes of interest for the mediation analysis were pain-related disability, pain, fear-avoidance beliefs, pain catastrophizing, and fear of movement and (re)injury (kinesiophobia).

Pain-related disability was measured with the Pain Disability Index (PDI), a 7-item questionnaire with a range of 0–70 points that assesses to what extent daily activities are disrupted by pain. The questions comprise aspects of family and home responsibilities, recreation, social activity, occupation, sexual behaviour, self care, and life-support activities. We used a Dutch language version, which has proven to be valid and reliable in patients with musculoskeletal pain [19].

We measured pain on a visual analogue scale (VAS-pain), a widely used, reliable and valid tool for the assessment of pain [20]. A score of 1 equals “no pain”, whereas 10 means “worst imaginable pain”.

The Fear-Avoidance Beliefs Questionnaire (FABQ) measures patients’ beliefs about how their pain influences work and physical activities [21,22]. The 16-item questionnaire has good psychometric properties [23] and the scores range from 0–42 points for the work subscale and from 0–24 for the physical activities subscale. We used the Dutch language version as translated by Vendrig et al. [22].

For measuring pain catastrophizing, we used the Dutch language Pain Catastrophizing Scale (PCS), a 13-item questionnaire with questions about the three aspects of catastrophizing: rumination, magnification and helplessness [24]. The PCS is one of the most widely used instruments for measuring catastrophic thinking related to pain and has good psychometric properties [25,26]. The PCS ranges from 0–52 points, with its subscales rumination, magnification and helplessness ranging from 0–16, 0–12, and 0–24 respectively.

We measured fear of movement and (re)injury using the Tampa Scale for Kinesiophobia-11 (TSK-11) [27], a brief, reliable and valid measure [28], with a score ranging from 11–44 points. We used the Dutch language version as translated by Vlaeyen et al.

### Data analysis

Because of the exploratory nature of this particular study, we did not abide to the randomization protocol and analysed all patients according to the treatment they received from the beginning of the trial.

**Fig 2:**
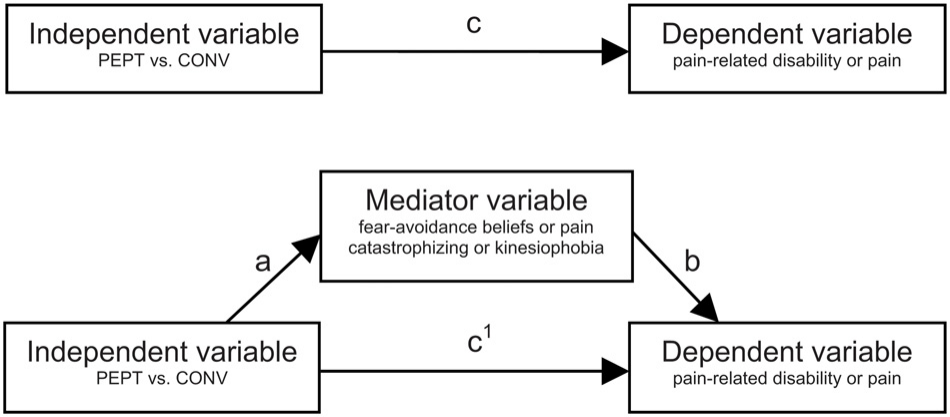
Model for the mediation analysis. This model was used to analyse potential mediation between the independent variable (Pain Exposure Physical Therapy versus conventional treatment), the mediator variable (fear-avoidance beliefs or pain catastrophizing or kinesiophobia), and the dependent variable (pain-related disability or pain) [30]. PEPT = Pain Exposure Physical Therapy. CONV = conventional treatment.

We used descriptive analyses to describe patient characteristics. Fig 2 shows the model we used to analyse potential mediation between the independent variable treatment (PEPT or conventional treatment), the mediators (fear-avoidance beliefs, pain catastrophizing, or kinesiophobia), and the dependent variables (pain-related disability or pain).

According to the steps described by Baron and Kenny [29], we planned to perform three analyses, using linear mixed models. First, we estimated the total effect of treatment (independent variable) on the dependent variables pain-related disability or pain (Fig 2, arrow c). Second, we estimated the effect of treatment on the potential mediators (Fig 2, arrow a). Only if both effects (between-group differences) were statistically significant, using alpha levels of up to 0.10 due to the exploratory nature of this study, we performed the third analysis, in which we regressed the dependent variables on both the mediators and the independent variable. For mediation to be present, the mediator had to be significantly related to the outcome (path b) and the direct effect of the independent variable on the outcome (arrow c^1^) had to be smaller than the total effect (arrow c) [30]. On the basis of the standardised coefficients, we also planned to assess the proportion of mediated effect: (c—c^1^)/c x 100% [30].

We used linear mixed models with unstructured repeated covariance, treatment and measurement in time as factors and outcome at baseline as covariate, to determine the between-group differences on the dependent variables over time. Because 26.8% of the patients switched groups prior to treatment initiation, shifts in baseline characteristics occurred. Therefore, we added these as potential confounders (affected extremity and dominant side affected as factors, and time since inciting event as covariate) to the analysis. If they affected the between-group estimates by more than 10%, we considered them as confounders and added them to the models [31]. Linear mixed models deal with missing data by predicting the best-fitting line for each patient without data imputation. We performed the analyses with SPSS 20.0.

### Ethics statement

The Ethics Committee on Research Involving Human Subjects of Arnhem and Nijmegen, The Netherlands (CMO Regio Arnhem-Nijmegen), approved this study. The study is registered at www.clinicaltrials.gov, NCT00817128, and at www.trialregister.nl, NTR 2090.

All participants provided both oral and written informed consent prior to data collection.

## Results

### Participants

Between January 2009 and June 2011, 56 patients were included in the PEPTOC trial. Thirty-five patients received Pain Exposure Physical Therapy and 21 patients received conventional treatment. Table 1 shows the baseline characteristics according to the treatment they received. At first glance, the CONV group contained relatively more patients with an affected upper extremity and the dominant side was relatively more often affected in this group. Also, the mean time since inciting event and the dispersion appeared different between groups. Although the differences in baseline characteristics were not significant between groups, the variables “affected extremity”, “dominant side affected”, and “time since inciting event” did alter the between-group estimates by more than 10% and we therefore added them as confounders to the linear mixed models [31].

**Table 1:** Baseline characteristics of the participants.

Baseline characteristics	Overall (n = 56)	PEPT (n = 35)	CONV (n = 21)
**Gender (female)**	45 (80.4%)	29 (82.9%)	16 (76.2%)
**Age (years)** *	44.3 (16.6)	43.1 (16.9)	46.2 (16.5)
**Affected extremity (upper)**	37 (66.1%)	20 (57.1%)	17 (81.0%)
**Dominant side affected**	32 (57.1%)	17 (48.6%)	15 (71.4%)
**Time since event (months)** †	6.0 (4.0–9.0)	6.0 (4.0–8.0)	6.0 (5.0–10.5)

* Mean (SD).

^†^ Median (interquartile range).

PEPT = Pain Exposure Physical Therapy. CONV = conventional treatment.

### Disability and pain (step 1)

**Fig 3:**
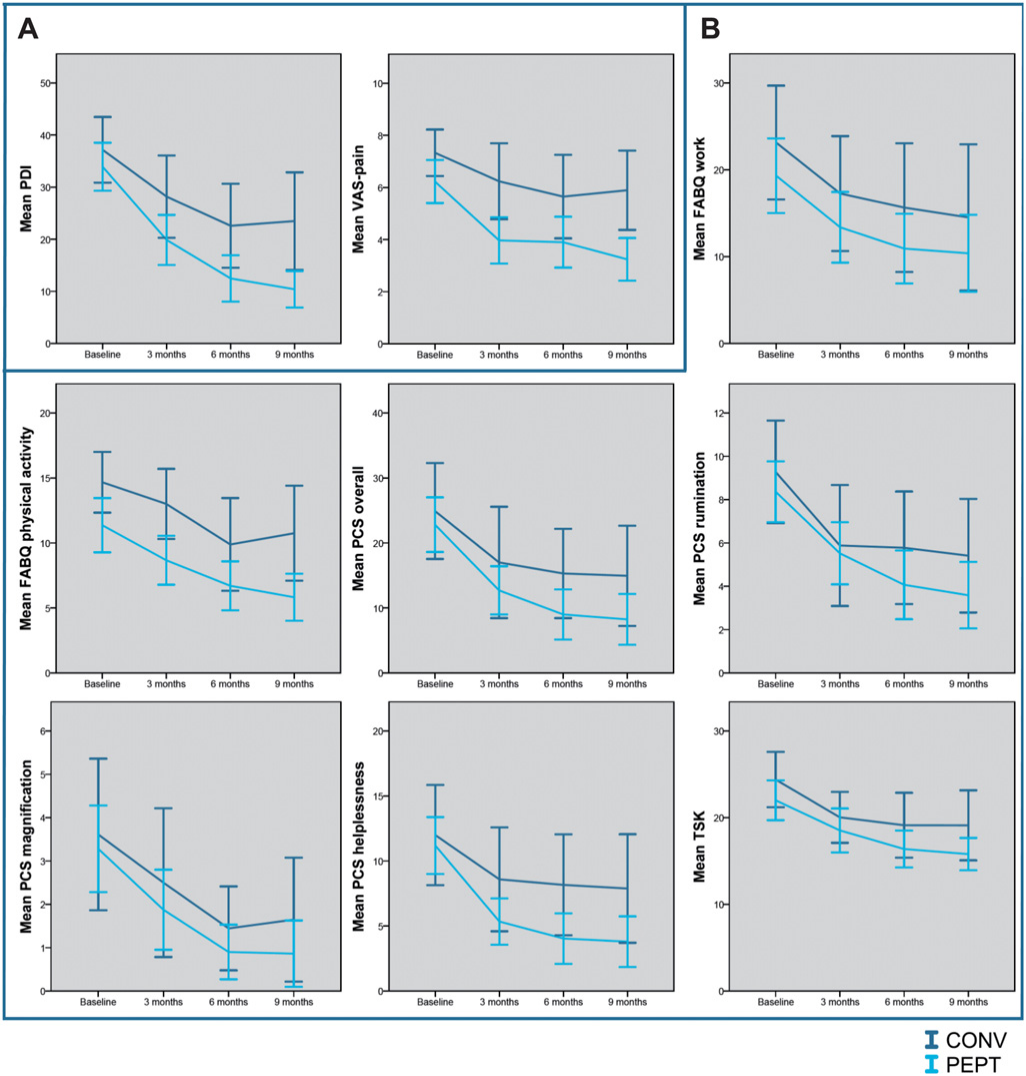
Outcomes for both treatment groups at baseline and at 3, 6, and 9 months follow-up. Panel A: dependent variables pain-related disability and pain. Panel B: potential mediators fear-avoidance beliefs, pain catastrophizing, and kinesiophobia. PDI = Pain Disability Index. VAS = Visual Analogue Scale. FABQ = Fear-Avoidance Beliefs Questionnaire. PCS = Pain Catastrophizing Scale. TSK = Tampa Scale for Kinesiophobia. CONV = conventional treatment. PEPT = Pain Exposure Physical Therapy.

Disability and pain improved significantly in both groups, as is shown in Fig 3 and Table 2. Disability decreased with 69% (mean improvement 23.9; 95% CI 18.7 to 29.1), and there was a pain reduction of 57% (mean improvement 2.9; 95% CI 1.9 to 3.9) for the patients in the PEPT group. In the CONV group, disability decreased with 37% (mean improvement 16.0; 95% CI 8.0 to 24.1), and pain decreased with 23% (mean improvement 1.4; 95% CI 0.4 to 2.5).

**Table 2:** Descriptives of the dependent variables and the mediator variables.

Dependent variable	Baseline	3 months	6 months	9 months
**Pain Disability Index (0–70)**				
**PEPT (n = 33)**	33.91 (13.00)	19.87 (13.56)	12.48 (11.96)	10.40 (9.54)
**CONV (n = 19)**	37.17 (13.06)	28.18 (16.38)	22.58 (16.23)	23.48 (18.81)
**VAS-pain (1–10)**				
**PEPT (n = 35)**	6.23 (2.40)	3.97 (2.47)	3.90 (2.62)	3.24 (2.31)
**CONV (n = 21)**	7.33 (1.96)	6.24 (3.21)	5.65 (3.42)	5.89 (3.16)
**Mediator variable**	**Baseline**	**3 months**	**6 months**	**9 months**
**FABQ work (0–42)**				
**PEPT (n = 32)**	19.32 (11.72)	13.38 (11.29)	10.92 (10.38)	10.38 (11.65)
**CONV (n = 18)**	23.14 (13.18)	17.26 (12.86)	15.65 (13.90)	14.51 (13.94)
**FABQ physical activity (0–24)**				
**PEPT (n = 33)**	11.36 (5.88)	8.67 (5.31)	6.70 (5.05)	5.82 (4.85)
**CONV (n = 18)**	14.67 (4.70)	13.00 (5.24)	9.88 (6.95)	10.75 (6.86)
**Pain Catastrophizing Scale overall (0–52)**				
**PEPT (n = 32)**	22.81 (11.65)	12.70 (10.31)	9.00 (10.32)	8.24 (10.29)
**CONV (n = 18)**	24.91 (14.83)	17.00 (16.67)	15.30 (13.81)	14.94 (15.00)
**PCS rumination (0–16)**				
**PEPT (n = 32)**	8.36 (3.90)	5.52 (3.98)	4.07 (4.25)	3.59 (4.03)
**CONV (n = 18)**	9.28 (4.76)	5.88 (5.43)	5.78 (5.22)	5.41 (5.10)
**PCS magnification (0–12)**				
**PEPT (n = 32)**	3.28 (2.77)	1.88 (2.56)	0.90 (1.69)	0.86 (2.01)
**CONV (n = 18)**	3.61 (3.52)	2.50 (3.45)	1.44 (1.95)	1.65 (2.78)
**PCS helplessness (0–24)**				
**PEPT (n = 32)**	11.19 (6.06)	5.34 (4.94)	4.03 (5.20)	3.79 (5.12)
**CONV (n = 18)**	11.99 (7.75)	8.58 (8.03)	8.16 (7.81)	7.88 (8.12)
**Tampa Scale for Kinesiophobia (11–44)**				
**PEPT (n = 32)**	22.00 (6.37)	18.53 (7.03)	16.38 (5.59)	15.79 (5.07)
**CONV (n = 19)**	24.39 (6.42)	20.03 (6.10)	19.13 (7.02)	19.11 (8.12)

All data are mean (SD).

PEPT = Pain Exposure Physical Therapy. CONV = conventional treatment. VAS = Visual Analogue Scale. FABQ = Fear-Avoidance Beliefs Questionnaire. PCS = Pain Catastrophizing Scale.

The analysis showed a significant total effect of PEPT on disability, with an estimated mean between-group difference of 7.8 points (95% CI 1.1–14.5; p = 0.02) in favour of PEPT. Pain improved on average 1.8 points more in the PEPT group than in de CONV group (95% CI 0.4–3.2; p = 0.01) (Table 3) after adjusting for confounding.

**Table 3:** Mediation analysis with estimated group differences based on linear mixed models*.

	Dependent variable	Independent variable	Estimated group difference (95% CI)	p-value
**Path c**	Pain Disability Index	PEPT vs. CONV	7.77 (1.09 to 14.45)	0.02
	VAS-pain	PEPT vs. CONV	1.83 (0.44 to 3.23)	0.01
**Path a**	FABQ work	PEPT vs. CONV	2.27 (-2.47 to 7.02)	0.34
	FABQ physical activity	PEPT vs. CONV	2.29 (-0.78 to 5.35)	0.14
	Pain Catastrophizing Scale overall	PEPT vs. CONV	1.72 (-3.31 to 6.75)	0.44
	PCS rumination	PEPT vs. CONV	0.03 (-2.04 to 2.10)	0.92
	PCS magnification	PEPT vs. CONV	0.00 (-0.88 to 0.89)	0.97
	PCS helplessness	PEPT vs. CONV	1.67 (-1.09 to 4.43)	0.17
	Tampa Scale for Kinesiophobia	PEPT vs. CONV	1.01 (-1.74 to 3.76)	0.68

*Linear mixed models with unstructured repeated covariance, treatment and measurement in time as factors and outcome at baseline as covariate, corrected for “extremity”, “dominant side affected”, and “time since inciting event” as confounding variables.

PEPT = Pain Exposure Physical Therapy. CONV = conventional treatment. VAS = Visual Analogue Scale. FABQ = Fear-Avoidance Beliefs Questionnaire. PCS = Pain Catastrophizing Scale.

### Fear-avoidance beliefs, catastrophizing, and kinesiophobia (step 2)

Fear-avoidance beliefs, catastrophizing, and kinesiophobia decreased significantly in both groups (Fig 3, Table 2). In the PEPT group, the score on the FABQ work and FABQ physical activities decreased with 46% (mean improvement 8.7; 95% CI 4.3 to 13.2) and 49% (mean improvement 5.1; 95% CI 2.4 to 18.6) respectively, pain catastrophizing decreased with 64% (mean improvement 13.8; 95% CI 8.9 to 18.6), and kinesiophobia with 56% (mean improvement 5.7; 95% CI 3.4 to 8.0). Patients in the CONV group experienced a decrease in the FABQ work and physical activities scores of 37% (mean improvement 6.0; 95% CI -2.2 to 14.3; n.s.) and 27% (mean improvement 5.6; 95% CI 0.3 to 10.9) respectively. Pain catastrophizing decreased with 40% (mean improvement 12.8; 95% CI 7.8 to 17.3) and kinesiophobia with 39% (mean improvement 6.1; 95% CI 2.8 to 9.4). However, significant between-group differences were found for neither the three potential mediators, nor their subscales, using alpha levels of 0.05 and 0.10 (Table 3).

### Mediation (step 3)

Because there were no significant effects of treatment on pain-related fears, we did not perform step 3 of the mediation analysis, nor could we assess the proportion of the mediated effect.

## Discussion

### Statement of principal findings

With this exploratory study on the working mechanism of Pain Exposure Physical Therapy, we found that in this population the effect of PEPT, compared to conventional treatment, on disability and pain was not mediated by pain-related fears. We also found that in both treatment groups pain-related fears improved significantly without primarily focusing at fear reduction. According to this study however, reduction of fear does not explain the working mechanism of PEPT beyond conventional treatment.

### Meaning of the study

In contrast to standard physical therapy and other conventional treatment modalities [16], PEPT does not primarily focus on reducing pain, but on reducing disability and improving motor activities. PEPT is based on the supposition that limited use or even non-use of an extremity, whether or not caused by fear of movement or pain, can lead to disease deterioration [9]. Patients’ behaviour towards pain can be an important aspect in experienced physical impairments [32]. PEPT interferes with disuse or non-use by directly stimulating motor activities and unrestricted use of the affected extremity in daily life. By forcing the extremity to take part in daily activities, it becomes part of the functioning body and the formerly disrupted body schema.

In patients with CRPS-1, shrinkage of the cortical representation of the affected extremity in the primary somatosensory cortex has been observed [33]. There is a miscommunication between the brain and the extremity and the degree of cortical reorganisation is directly related to pain intensity [3]. Cortical reorganisation can be modified by behavioural interventions that provide correct feedback to the brain [34] and restore the communication. Analgesics and other types of treatment that could interfere with this communication should be avoided. Therefore, all medication aimed at CRPS-1 are stopped prior to treatment with PEPT. Maihofner et al. showed that cortical reorganisation can be reversed after effective treatment of CRPS-1 [35]. Performing normal activities and realizing this can be done without harm can be a trigger for cortical re-reorganisation, which consequently can lead to a decrease of pain [3].

Involving spouses in the treatment of CRPS-1 is an important component of the treatment. Not only should spouses know what patients are dealing with and should they ensure that the patients perform their exercises and support them, it is important that spouses ignore patients’ expressions of pain. It is demonstrated that spouses who reinforce pain can cause a 2.5-fold increase in patients’ brain response to pain, measured with electroencephalographic potentials, compared to spouses who ignore pain or punish the patients for expressing pain [34].

Our exploratory study showed that patients with CRPS-1 can be effectively treated, even though treatment is not primarily focused on reduction of pain or fear. However, since we found no between-group difference in reduction of fear, we cannot conclude that this treatment effect was caused by a reduction in pain-related fears.

A number of reasons can be identified for why we did not find a difference in decrease of fear between PEPT and CONV. The most obvious reason is that there is no difference in fear reduction between the two treatments. It is possible that both treatments reduce fear in their own way. With conventional treatment, there is definitely some attention for the fear patients experience; patients do not have to cross their own “boundaries”. Reinforcement of success experiences is used to show that the patients do not have to fear movement nor pain. PEPT, on the other hand, enhances the patients’ self-efficacy and stresses the patients to ignore their pain and use their affected limb despite possibly eliciting pain. As a result of their success experiences, this could explain a reduction of fear in patients treated with PEPT. In both cases, reduction of fear could be related to the experience that no harm is done. Apart from an effect of treatment, the reduction of fear in both groups could be due to a favourable natural course [36]. When pain is reduced, either following treatment or naturally, fear can be reduced even further. Paying attention to the patient and their condition may be beneficial, as is regaining confidence in a positive outcome.

In the multiple single-case study by Van de Meent et al., using a similar cohort, there was however no reduction in kinesiophobia during a baseline period of three months prior to treatment initiation [15]. This suggests that the improvement in pain-related fears we found in both groups resulted from treatment rather than a natural course.

### Strengths and weaknesses of the study

We did not abide to the randomization protocol. Because with this particular study we wanted to explore the working mechanism of PEPT, we looked at the sole effects of both treatments. Therefore, we analysed the patients according to the treatment they received from the beginning of the trial, while adjusting for confounding. The sample size calculation of the trial was consequently based on detecting differences in clinical effectiveness between the two interventions, rather than on mediation effects. As a consequence, the sample size may not have been adequate to identify mediation effects, which may have led to a type II error. To reduce the chance of a type II error and because of the exploratory nature of this study, we also interpreted the results of the analysis using an alpha of 0.10. However, this did not change our conclusion.

One of the inclusion criteria was first assessment between 3 and 24 months after the inciting event, yet the median duration of complaints for the participants in this study was rather short (6 months). Only 12.5% of the patients reported at our outpatient clinic ≥12 months after the inciting event. This reduces the generalizability of our results to a population with more chronic patients.

During the screening process, we did not systematically check for psychological or psychiatric comorbidities by means of questionnaires or psychological evaluation. Possible differences could add some explanation to our data. However, we did perform a thorough history taking in all patients during screening, which did not reveal the presence of such comorbidities. Throughout the trial, a psychologist was available in case there were concerns about the psychological condition of patients.

Because this is the first study that explored possible mediation of pain-related fears in treating patients with CRPS-1 with PEPT, there are no other studies to compare our results with. However, there are several trials on treatment modalities focusing on reduction of fear in patients with CRPS-1. One of these trials (De Jong et al. [11]) studied graded exposure therapy (GEXP), a form of cognitive behavioural treatment that is aimed at reducing pain-related fears. In approximately 20 treatment sessions, performed by a behavioural therapist and a rehabilitation specialist, patients were gradually exposed to situations they had identified as dangerous or threatening. De Jong et al. included only females (n = 8), with a mean duration of complaints of 3.0 years (at least 6 months) and patients had to report substantial kinesiophobia (TSK>39 on the 17-item questionnaire; corresponding to TSK>25 on the 11-item questionnaire) at baseline. The results of this single-case experimental trial showed that GEXP was successful in decreasing pain-related fear, pain intensity, and disability in female patients [11].

Although we cannot compare the results of both trials, because we included patients with a relatively short history, disregarding their levels of fear, we can state that it appears that in an average population with a relatively short duration of complaints, CRPS-1 can be effectively treated, without primarily focusing on fear, thus avoiding long and costly treatment. After thorough education that pain is not a sign of injury and moving is not harmful but rather promotes quick improvement, it is possible to reduce disability and pain in a safe and effective manner.

Because, in contrast to PEPT, GEXP applies graded exposure, the duration of treatment is longer (10 to 20 sessions with GEXP [8,11], compared to a maximum of 5 treatment sessions with PEPT [17]), probably making it more costly than PEPT.

### Unanswered questions and future research

It should be emphasized that our study findings need to be replicated in other, preferably larger, studies to elucidate the potential role of pain-related fears as mediators of the effects of PEPT on pain and disability.

Furthermore, it would be interesting to systematically compare graded exposure therapy and Pain Exposure Physical Therapy and see which of the two treatment modalities is more effective and whether patient characteristics, such as disease duration and levels of fear, influence their effectiveness.

In our trial, patients did not report excessive levels of pain-related fears, compared to other patients with CRPS-1 (mean TSK = 24 [9]), soft tissue injury (mean PCS = 28 [37]), low back pain (FABQ work >34 [38], FABQ physical activity >14 [39], TSK >24[27], mean TSK = 43 [40]), or fibromyalgia (mean TSK = 37 [40]). It could be possible that patients with too much fear or chronic complaints do not benefit from PEPT because they lack internal motivation or feelings of self-efficacy. PEPT might even provoke fear in these patients, preventing them from committing to the treatment. Graded exposure might be a better approach for such patients.

In conclusion, our analysis showed that there was no indication that reduction of disability and pain by PEPT, in comparison to conventional treatment, was mediated by pain-related fears, regardless of whether the reduction of fear was caused by treatment or by any other factor. Hence, this seems to indicate that pain-related fears do not have to be treated specifically in order to reduce disability and pain effectively in patients with CRPS-1.
